# Classification of Electroencephalography Motor Execution Signals Using a Hybrid Neural Network Based on Instantaneous Frequency and Amplitude Obtained via Empirical Wavelet Transform

**DOI:** 10.3390/s25113284

**Published:** 2025-05-23

**Authors:** Patryk Zych, Kacper Filipek, Agata Mrozek-Czajkowska, Piotr Kuwałek

**Affiliations:** 1 Institute of Communication and Computer Networks, Poznan University of Technology, 60-965 Poznan, Poland; patryk.zych@student.put.poznan.pl; 2 Faculty of Mechanical Engineering, Poznan University of Technology, 60-965 Poznan, Poland; kacper.filipek@student.put.poznan.pl; 3 Institute of Material Technology, Poznan University of Technology, 60-965 Poznan, Poland; agata.mrozek-czajkowska@put.poznan.pl; 4 Institute of Electrical Engineering and Electronics, Poznan University of Technology, 60-965 Poznan, Poland

**Keywords:** brain–computer interface, electroencephalography, motor execution, hand gesture, empirical wavelet transform, machine learning, classification

## Abstract

Brain–computer interfaces (BCIs) have garnered significant interest due to their potential to enable communication and control for individuals with limited or no ability to interact with technologies in a conventional way. By applying electrical signals generated by brain cells, BCIs eliminate the need for physical interaction with external devices. This study investigates the performance of traditional classifiers—specifically, linear discriminant analysis (LDA) and support vector machines (SVMs)—in comparison with a hybrid neural network model for EEG-based gesture classification. The dataset comprised EEG recordings of seven distinct gestures performed by 33 participants. Binary classification tasks were conducted using both raw windowed EEG signals and features extracted via bandpower and the empirical wavelet transform (EWT). The hybrid neural network architecture demonstrated higher classification accuracy compared to the standard classifiers. These findings suggest that combining featuring extraction with deep learning models offers a promising approach for improving EEG gesture recognition in BCI systems.

## 1. Introduction

Over the years, brain–computer interfaces (BCIs) have gained increasing interest. This communication link supports individuals who are unable to use everyday technologies in a conventional way, providing them with essential accessibility and interaction capabilities. It assists individuals who, as a result of various incidents, have experienced spinal cord injuries, brain trauma, or strokes, as well as those affected by diseases that impair the nervous and muscular systems, such as amyotrophic lateral sclerosis (ALS), multiple sclerosis, or muscular dystrophies [1]. The need for physical, manual interaction with external devices is eliminated by utilizing the user’s brain activity [2,3]. This allows brain–computer interfaces to be applied in various medical and rehabilitation applications, with their functionality limited only by the patient’s needs and the ability to generate the appropriate EEG signal pattern. BCIs are used in rehabilitation systems [2,4,5,6,7] and they enable communication with the external world [8,9,10], control of prosthetic limbs [11,12,13,14], wheelchairs [15,16,17], and hearing aids [18], and they provide people with support in pursuing hobbies and passions, such as painting [19]. Their primary classification is based on invasiveness, distinguishing between invasive and non-invasive BCIs. The first type allows for signal measurement inside the skull, requiring surgical intervention. Implanted electrodes measure brain activity beneath the skull and within the cerebral cortex. A less invasive method, electrocorticography (ECoG), records neural signals from between the cortical surface and the skull. Invasive brain–computer interfaces provide higher spatial resolution compared to non-invasive ones, which rely on devices that do not directly interfere with the patient’s body. In the case of non-invasive BCIs, brain signal measurements are primarily conducted using magnetoencephalography (MEG), electroencephalography (EEG), functional magnetic resonance imaging (fMRI), positron emission tomography (PET), and functional near-infrared spectroscopy (fNIRS). Each of these techniques is based on different physical principles and varies in terms of equipment size, cost, measurement method, and spatial and temporal resolution [2,3,17,20]. Among these, the most commonly used method for data acquisition in non-invasive brain–computer interfaces is EEG. Modern BCIs are based on the principle that each brain region is associated with a specific function of the body. Popular signals include the following: Steady-State Visual Evoked Potentials (SSVEPs), which can be detected in the occipital lobe; P300 potential (also known as the P300 wave), which appears in the parietal lobe; Event-Related Desynchronization (ERD) and Event-Related Synchronization (ERS), which occur primarily in sensorimotor areas [3,21,22,23,24]. ERD/ERS are therefore related to the motor activity of the extremities. Movement of any limb first leads to desynchronization, meaning a decrease in mu and beta oscillations, which is observed during the body’s preparation for movement. After the actual movement is performed, these oscillations increase again (synchronization). In the case of upper limbs, this relationship is visible in the contralateral hemisphere of the brain relative to the moving extremity (i.e., for the right hand, it is the left hemisphere, and for the left hand, the right hemisphere). For the lower limbs, however, the brain region where these changes occur is located in the central area. Interestingly, this phenomenon also occurs during motor imagery (MI) [24,25,26,27]. Brain–computer interfaces based on motor execution (ME) or imagery for detecting ERD and ERS mainly use bandpower as a feature [28,29,30]. However, in recent years, researchers have focused their attention on identifying additional features that can enhance the effectiveness of classification. Among the most prominent approaches are the use of the Spatial Covariance Matrix (SCM), statistical and spatial features, wavelet coefficients at various decomposition levels, higher-order statistics (HOSs), Hjorth parameters, fractal dimensions, and their combinations [31,32,33]. The research that utilized instantaneous amplitude (IA) and instantaneous frequency (IF) , along with HOS derived from them, is worth noting; this research yielded satisfactory results in motor imagery classification [34].

The key components of BCIs are preprocessing, feature extraction, and classification [34]. Proper signal preparation is essential for eliminating noise or artifacts and isolating the desired signal bands. This can be achieved through appropriate filtering or other methods such as Principal Component Analysis (PCA) and independent component analysis (ICA) [35,36,37]. It is also worth noting that PCA and ICA are also used as a feature reduction techniques [38,39]. In addition, independent components (ICs) corresponding to electrical sources identified by ICA can be used as a features to support ERD/ERS classification. In general, ICs representing time–frequency domain activity contribute more significantly to classification accuracy than other components [40,41]. Effective approaches also include combining ICA with Powers Spectral Density (PSD) for feature extraction and employing supervised independent component analysis algorithms for MI tasks [42,43]. Feature extraction and classification enable the automated and desired operation of a device based on expected actions. Among the available feature extraction methods, notable ones include Fourier Transform (FT), autoregressive (AR) models, common spatial patterns (CSPs), statistical clustering, iterative spatio-spectral patterns learning (ISSPL), and optimal allocation [44,45,46,47,48,49]. Despite their extensive use in motor-related applications, these methods also come with various drawbacks, such as the lack of temporal information for EEG signals, susceptibility to noise, and a low success rate in classification [35,50]. In recent years, signal decomposition (SD) methods have been increasingly used for the same issue. These methods operate by generating multiple sub-band signals. Among them are wavelet transform (WT), wavelet packet decomposition (WPD), discrete wavelet transform (DWT), continuous wavelet transform (CWT), empirical mode decomposition (EMD), and empirical wavelet transform (EWT) [34,35,45,50,51,52]. Unlike traditional wavelet-based approaches such as DWT or CWT, the EWT method enables efficient extraction of time-varying components by using empirically determined wavelet filter parameters. This avoids unnecessary data redundancy and improves the precision of separating nonstationary and multi-component signals, thereby minimizing band mixing artifacts. In contrast, classical wavelet methods apply fixed filter parameters over time, which can lead to erroneous segmentation, such as splitting useful signal components or separating channels that contain no meaningful information. Findings across various disciplines indicate that EWT offers significant advantages over classical DWT, especially in the context of nonstationary signals such as EEG data [53]. Prior studies have shown that EWT achieves improved performance in signal decomposition and feature extraction compared to traditional wavelet-based approaches. Moreover, the existing literature suggests that EMD offers improved segmentation capabilities relative to DWT [54], while other work has demonstrated that EWT outperforms EMD in specific applications [55], further reinforcing its utility as a robust tool for analyzing complex, time-varying signals. EWT has been successfully applied in the medical domain, including signal processing (such as EEG and ECG), medical image analysis, and anatomical structures segmentation [55,56,57,58]. One noteworthy application is its use in motor imagery-related research [34,35,50,51]. Therefore, an area worthy of further investigation is its application in the motor execution paradigm, which could be useful in BCI systems supporting rehabilitation. EWT is an extended version of empirical mode decomposition used for analyzing non-linear and nonstationary signals. It enables multiresolution analysis (MRA) by employing an adaptive wavelet partitioning scheme, decomposing the signal into distinct sub-bands based on the information it carries [55]. Due to the nature of its operation, the EWT can produce inappropriate segmentations in the presence of high noise, which may negatively impact the performance of the machine learning method applied. One approach to mitigate this issue is the Enhanced Empirical Wavelet Transform (EEWT), which improves signal decomposition (particularly in non-stationary signals) through spectrum pre-regularization and segmentation based on the signal’s spectral envelope. However, EEWT is computationally more complex and requires careful hyperparameter optimization, making it challenging to implement on hardware with limited processing capabilities.

Gesture recognition using EEG signals requires advanced time–series analysis methods. Traditional approaches rely on feature engineering-based algorithms and classical statistical models [59]. Among the classifiers commonly used in ME and MI tasks that yield satisfactory results, linear discriminant analysis (LDA) [6,60,61,62] and support vector machine (SVM) [6,35,51,61,62,63] are notable. Furthermore, their extensions, such as least squares support vector machine (LS-SVM) [34,34] and time-varying linear discriminant analysis (TVLDA) [29], are also used. However, advancements in deep learning have enabled the automatic extraction of relevant information without the need for manual feature extraction [64]. In neural networks designed for EEG signals, the use of convolutional layers is particularly important to analyze for the spatial structure of the data. Convolutional neural networks (CNNs) enable the automatic detection of local patterns in brain signals, which is crucial for identifying the distinctive features of a given gesture [65]. The application of long short-term memory (LSTM) networks enables the capture of long-term dependencies over time, which is essential for the sequential nature of EEG signals [66,67,68]. To ensure effective gesture classification, the appropriate selection of dense layers and the output layer is crucial [69]. Despite significant progress being made in applying neural networks to EEG analysis, several challenges require further research. One of the main issues is the limited availability of data, which can lead to model overfitting [70]. Another challenge is model personalization, as brain signal patterns can vary significantly between users, making classification more difficult [71,72]. The need to develop adaptive methods that allow effective adjustment of the model to individual users is becoming increasingly important in the context of real-world BCI applications [73,74].

The effectiveness of gesture recognition can be enhanced by incorporating other electrophysiological signals, such as electromyography (EMG) [75]. However, this approach complicates the measurement infrastructure [76,77]. A simpler and more convenient alternative is to process brain signals directly, which can be recorded using widely available EEG headsets. When detecting movement in specific limbs (e.g., left arm vs. right arm or arm vs. leg), motion recognition may prove easier for traditional classifiers. This is due to brain physiology and the observable dominance of potentials in specific electrode placements (e.g., for right-hand movement, ERD/ERS can be observed in the left hemisphere, leading to detectable changes in channels such as C3). In contrast, the detection of more complex gestures has not yet been sufficiently explored in the context of non-invasive signal acquisition methods, necessitating extensive further research.

In this article, we compare the use of artificial neural networks (ANNs) with standard classifiers for detecting specific gestures. In addition, we evaluate the effectiveness of ANNs using both filtered signals and extracted features as input. The aim of this work is to develop an algorithm capable of detecting specific gestures, which will contribute to the advancement of non-invasive BCI systems designed to support the rehabilitation of individuals experiencing neuromuscular dysfunction, such as those recovering from a stroke.

## 2. Materials and Methods

The article presents a comparison of classification using the two most common classifiers in motor imagery applications (LDA and SVM) and the results achieved with a hybrid neural network (HNN) that integrates CNN and LSTM, allowing efficient processing of the spatial–temporal aspects of EEG signals [75,78]. Furthermore, the influence of signal processing and feature extraction on the results will be analyzed. All classification tools will be trained based on the preprocessed signal, along with feature extraction. For the neural network, training will also be conducted on data without extracted features to enable a comparison.

### 2.1. Dataset Description

In the presented study, the available online dataset [79] was utilized. The experiment involved 33 non-disabled subjects without any neuromuscular dysfunction. This group consisted of 17 right-handed men, 10 right-handed women, 3 left-handed men, and 3 left-handed women, with an average age of 25 ± 5.5 years. Among all participants, 24 were familiar with and had experience using BCIs. The measurement setup is an electroencephalograph. Brain signals acquisition was performed using the OpenBCI Ultracortex “MARK IV” headset, which included the following measurement electrodes: F3, F4, C3, Cz, C4, P3, Pz, and P4. This configuration enabled coverage of the motor cortex, thus maximizing the registration of motor-related potentials. One of two reference electrodes serving as the reference and bias (ground with common-mode noise rejection) for EEG system was placed on each earlobe. All electrodes were placed according to the international 10-20 system. The sampling rate was 250 Hz. The experimental protocol was explained to the subjects beforehand. It consisted of performing six repetitions (the last two repetitions were performed while standing) of each of the seven gestures: large diameter, cut, medium diameter, open, power, prismatic pinch, and three-finger sphere grasp. Each trial was preceded by the display of the target gesture on a screen located 1.5 m from the participant, along with a simultaneous acoustic tone signaling the need to begin performing the indicated gesture for 5 s. After each trial, a 3 s rest interval followed. In total, 42 trials were conducted per subject. To minimize environmental interference and enhance signal quality, all experimental sessions were conducted in a noise-reduced room.

### 2.2. Preprocessing

**Figure 1:**
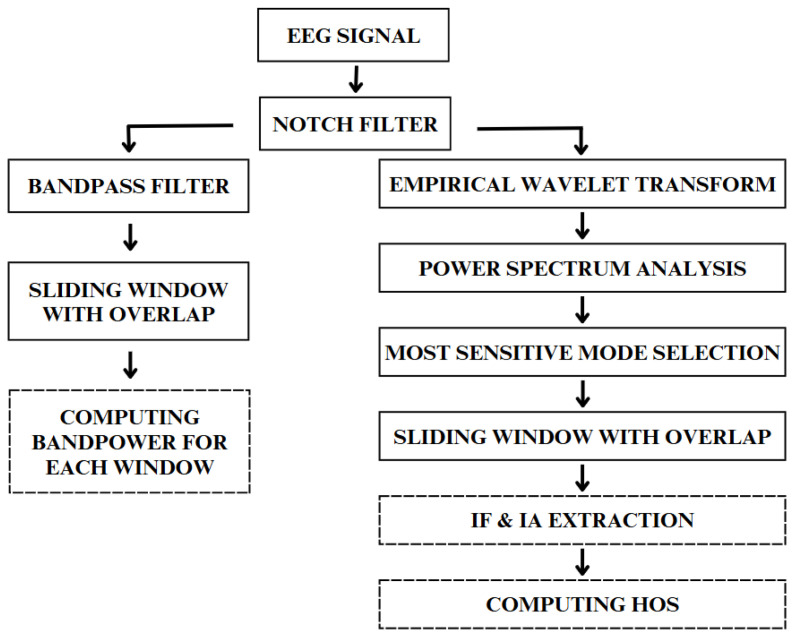
The individual preprocessing steps for both variants (first on the left, second on the right). Blocks with a dashed border indicate the feature extraction stage.

The EEG data in the dataset are represented in microvolts (μV). Initially, the signal was filtered using a 5th order Butterworth bandstop filter [80] to remove the 60 Hz component to prevent interference from power line noise. Then, two processing variants were considered (Figure 1), additionally involving the use of either all measurement channels or only C3, Cz, and C4. In the first variant, a frequency band from 8 to 25 Hz was extracted using a 5th order Butterworth bandpass filter, corresponding to the mu and beta rhythm ranges [78]. To increase the number of training samples, the signals were segmented using a sliding window with 60% overlap [75]. Finally, band power was computed for each window, serving as the feature for classifier training. It is important to emphasize that the quality of input data is crucial for the correct performance of both standard classifiers and neural network models, making preprocessing steps like filtering and segmentation essential for reliable model training and evaluation. In the second variant, after removing power line interference, the empirical wavelet transform method was applied to decompose the nonstationary signal into different modes, each adjusted around an explicit frequency to fulfill the properties of intrinsic mode functions (IMFs). This was achieved using filter banks obtained through a process that first computed the signal spectrum in the range of 0 to π using Fast Fourier Transform (FFT). Then, EWT boundary detection was performed (hyperparameters were optimally selected using brute force optimization method), followed by the application of empirical wavelets to all segmented spectra as bandpass filters [55]. Using the MATLAB 2024b library created by the author of EWT [81], the EEG signal was decomposed for each channel, empirically setting the number of modes to 10 per channel. For each mode, power spectrum analysis was performed using the Welch PSD method excluding frequencies up to 2 Hz, to detect the mode with the highest power concentration, indicating its highest sensitivity to the ME task. Based on the findings of [34], feature extraction focused on HOS derived from instantaneous frequency (IF) and instantaneous amplitude (IA). To extract IF and IA, the Hilbert–Huang Transform (HHT) approach [82] was applied to each sliding window with 60% overlap from the most sensitive mode. For each IMF in the first step, the Hilbert–Huang procedure computes the analytic signal:

(1) $$z_{i}\left(t\right)=x_{i}\left(t\right)+jH\left\{x_{i}\left(t\right)\right\},$$

where *H*$x_{i}$ is the Hilbert Transform of $x_{i}$ (data values in the set). Then, it expresses $z_{i}$ as

(2) $$z_{i}\left(t\right)=a_{i}\left(t\right)e^{j\theta_{i}\left(t\right)},$$

where *$a_{i}$*(*t*) is the instantaneous amplitude and $\theta_{i}$(t) is the instantaneous phase. Finally, it computes the instantaneous frequency

(3) $$\omega_{i}\left(t\right)\equiv \frac{d\theta_{i}\left(t\right)}{dt}.$$

For classification, four time domain feature vectors were extracted from the instantaneous components:

- Mean absolute deviation (MAD) measures statistical data distribution (4).
- Interquartile range (IQR) determines dataset distribution by excluding lower and upper quartiles (5) [83,84].
- Skewness (Sk) describes the asymmetry of data distribution around the mean (6).
- Kurtosis (Kt) quantifies data flatness compared to a Gaussian distribution; combined with skewness, this helps identify linear, stationary, and Gaussian anomalies in signals (7) [45].

(4) $$\mathrm{MAD}=\frac{1}{n}\sum_{i=1}^{n}\left|x_{i}-\bar{x}\right|,$$

(5) IQR=Q3−Q1,

(6) $$\mathrm{Sk}=\frac{\frac{1}{n}\sum_{i=1}^{n}{\left(x_{i}-\bar{x}\right)}^{3}}{{\left(\sqrt{\frac{1}{n}\sum_{i=1}^{n}{\left(x_{i}-\bar{x}\right)}^{2}}\right)}^{3}}$$

(7) $$\mathrm{Kt}=\frac{\frac{1}{n}\sum_{i=1}^{n}{\left(x_{i}-\bar{x}\right)}^{4}}{{\left(\frac{1}{n}\sum_{i=1}^{n}{\left(x_{i}-\bar{x}\right)}^{2}\right)}^{2}}$$

where *n* is the number of data values, $\bar{x}$ is average value of the dataset, *Q*1 (first quartile) is the median of the lower half of the dataset, and *Q*3 (third quartile) is median of the upper half of the dataset. The choice of these four features is supported by their increasing popularity in the analysis of biomedical signal, as well as by findings from MI detection studies, where their use has been shown to improve classification accuracy and enhance the stability of detection results across different MI tasks [34,45,83,84,85]. Therefore, it is worth investigating whether these features can yield similar benefits in the context of ME tasks.

### 2.3. Deep Learning Models

In this study, deep learning was employed to perform binary classification of EEG gesture data using both windowed signals and features derived from the computed band power and the empirical wavelet transform, specifically the instantaneous amplitude and frequency calculated from the same windows. While patient-oriented modeling proves more efficient in this case [86], our dataset was not sufficiently large to support personalized models. Therefore, we focused on developing a generalized model capable of capturing common neural features between subjects. For each classification task, a one-vs-rest (OvR) strategy was used, in which a dedicated model was trained to distinguish a single gesture class from all others. In order to increase reliability and generalizability, training and testing was carried out in a mixed dataset that represents the trials of a given gesture made by individual subjects. The deep learning architecture was designed to take advantage of the temporal and spatial structure of the input signal. The architecture in general consists of the following components:

- Input layer—input data in form of time–series matrices.
- Convolutional layers—a stack of two 1D convolutional layers (32 filters of size 5 and 64 filters of size 3, respectively) capture local temporal features from the multichannel signal.
- Pooling layers—max-pooling layers with a stride of 2 reduce the temporal dimension and help avoid overfitting [87].
- Bidirectional LSTM layer—a bidirectional long short-term memory (BiLSTM) layer with 64 units enables the model to learn long-range temporal dependencies in both forward and backward directions, which is crucial for modeling the dynamics of gestures [88].
- Dense layers—a fully connected layer with 64 neurons (ReLU activation) and an output layer with a single neuron (sigmoid activation) produce the binary prediction.

The model was trained with the 80/20 split dataset, utilizing the Adam optimizer and the binary cross-entropy loss function. Batch sizes ranging from 6 to 20 and the number of epochs per gesture were adjusted to optimize performance. Due to the inherent class imbalance in the OvR setting, the training set was balanced using RandomOverSampler from the "imbalanced-learn" package to avoid model bias toward the majority class [89]. Each model was evaluated using unseen trials of the target gesture as positive samples and gestures of all other classes as negative samples.

### 2.4. Performance Metrics

To evaluate the accuracy of the classification, the dataset was divided into training and test sets using the *k*-fold cross-validation method (in the case of LDA and SVM). The parameter *k* determines the number of equal-sized partitions into which the dataset is divided. A higher value of *k* results in more iterations and more partitions (which also means significantly more feature vectors in the training set compared to the test set) [90,91]. In this study, the parameter *k* was set to 5, which means that the dataset was divided into five equal partitions, with 80% used for training and 20% for testing. This split ensures a sufficiently large test partition for obtaining reliable results while maintaining a reasonable number of experiments, reducing computational time, and minimizing estimator variance [34]. Cross-validation was performed using a dataset of 1386 samples, each sample representing an individual’s execution of a particular gesture and comprising features derived from 61 time windows per EEG channel. Accuracy, representing the ratio of the number of correct assessments to the number of all assessments (8), and F1 score, calculated as the harmonic mean of the precision and recall scores (9), were used as statistical parameters to measure algorithm performance:

(8) $$\mathrm{accuracy}=\frac{\mathrm{TP}+\mathrm{TN}}{\mathrm{TP}+\mathrm{TN}+\mathrm{FP}+\mathrm{FN}},$$

(9) $$F1\;\mathrm{score}=\frac{\mathrm{TP}}{\mathrm{TP}+\frac{1}{2}\left(\mathrm{FP}+\mathrm{FN}\right)},$$

where TP (true positive) represents the number of correctly classified executions of the desired gesture. TN (true negative) refers to the number of correctly classified executions of any other gesture. FP (false positive) indicates the number of incorrectly classified executions of other gestures that were misclassified as the desired gesture. Conversely, FN (false negative) represents the number of incorrectly classified executions of the desired gesture, misclassified as one of the other gestures.

## 3. Results

The classifiers were trained using the OvR strategy based on the dataset studied in different configurations. This includes extracting channels C3, Cz, and C4, as well as evaluating the set of characteristics that considers IF and IA together and separately in the second variant. The best accuracy for the given variants and their combinations, which also achieved the highest F1 score in the studied dataset, is presented in Table 1 (the result in bold text indicates the highest score obtained in the entire study).

In addition, for comparison, the results of the remaining classifiers for the configuration that achieved the best performance are also included. During the study, it was observed that the best results were obtained when using signals from all EEG measurement channels. Therefore, information on the channels utilized was omitted from the variant description in Table 1. To compare the proposed hybrid model with traditional classifiers, Wilcoxon signed rank tests were performed using both the F1 score and the accuracy metrics in all gesture classes. The results indicate that the improvements achieved by the hybrid model are statistically significant (*p* < 0.05).

## 4. Discussion

The collected EEG dataset comprised seven gestures performed by 33 participants, each of whom completed six tests of each movement, with the last two repetitions performed while standing. However, this dataset presented significant limitations. In particular, only the signal associated with the execution of the gesture was recorded, without any temporal segments captured before or after the gesture. This required gesture detection to rely solely on the data recorded during its execution, thus necessitating a one-vs-rest strategy. Such an approach limits the ability to thoroughly analyze the dynamics of brain activity (e.g., it may omit the preparatory phase characterized by neural desynchronization or the post-movement physiological phenomenon of neural synchronization) and significantly complicates the ability of models to learn and recognize patterns. It is also important to note that the nature of EEG signals does not allow for typical interpolation or augmentation techniques, which forces deep learning models to rely solely on real data; this is problematic in this case due to the relatively small dataset. In addition, the spatial resolution of EEG is relatively low compared to other invasive methods. Significantly improved detection accuracy of individual finger movements is possible through the use of electrocorticography [29]. This is equivalent to making the entire task more challenging and making individual gestures more difficult. Furthermore, the dominant hand of the patients can also influence all of the results, potentially affecting the power of the generated signals, as well as the pattern of signal occurrence on specific measurement electrodes. The analysis included the use of basic classifiers such as LDA and SVM, as well as a complex CNN model with a bidirectional LSTM layer, trained on sequential data. An approach used the full sequence of 61 time windows without additional feature extraction. Another used a dataset consisting of features extracted from the data within each window. The tested approaches used band power (in the first variant) and feature extraction based on higher-order statistics derived from IA and IF parameters (in the second variant), which also reduced data dimensionality while preserving their characteristics. The highest accuracy and F1 score were achieved using the second variant, which applied higher-order statistics derived from both instantaneous frequency and instantaneous amplitude.

The highest obtained accuracy was equaled to 81.9%. The baseline for comparison was a study [75] using the identical dataset. In [75], an accuracy of 49.3% and an F1 score of 49.0% were reported. However, it should be noted that the approach presented in that article differs from the one compared here. In the present study, the focus was on creating a generalized algorithm to distinguish gesture data, while the other article involved tailoring a neural network model to each individual participant, which could ultimately have led to a higher average F1 score.

In the case of the authors of [34], who studied the motor imagery paradigm in five patients individually, their best results (accuracy 95.19%, F1 score was not provided) were achieved using the same HOS derived from IA. Their study was limited to only two MI tasks for the right hand and leg and did not cover as many gestures performed by the same limb. Given the physiological basis of ERD/ERS, detecting these events might have been easier for the classifier, at least because of the dominance of this phenomenon in different EEG channels.

Another example of using EEG signals to gesture recognition was research presented in [92]. The algorithm applied is NeuroGraps—a dual-stage deep learning framework. Two scenarios were considered—learning to distinguish between two gestures and learning to distinguish between four gestures. The number of gestures significantly influenced the accuracy obtained (68% for four gestures, 86% for two gestures). Learning was based not only on EMG, but was supported by EMG signals. In contrast, only the EEG signal was used during network testing. The accuracy obtained during the tests performed (89.1%) is comparable to a scenario in which two gestures were considered. Nevertheless, adding more gestures can significantly reduce the accuracy of the classification—therefore, it may be necessary to extend the signal set with EMG data. This phenomenon was also observed in a study [93] where a classification accuracy of 60.8–62.4% was achieved applying single-modal EEG. In the case of recognition based on the EEG signal, but with a pre-trained model using both the EEG and the EMG signals, an accuracy of 65.1–66.5% was obtained. Using both EEG and EMG signals for identification, the accuracy increased to a range of 85.93–87.49%. A comparable accuracy for classification based on both EEG and EMG signals (80.5%) was obtained in a study [94] in which dynamic elbow flexion–extension movements were performed under varying load conditions. However, research for optimal gesture recognition tools based on the EEG signal alone is important to minimize the impact of signal strength-dependent variability of the EMG signal, injury, or surgical interventions performed.

The variability of the accuracy obtained in the population was subject-specific. Within one model, the difference between the patient with the highest F1-score and the lowest was even 51%. F1-scores vary significantly between patients, suggesting that the model does not generalize equally effectively for all patients. The results suggest that individual differences between patients have a significant impact on the performance of the classifier. It may be recommended to personalize the model. For all the cases analyzed, the model was significantly more effective in classifying open, power, prismatic pinch, and three-finger sphere grasp gestures. The results obtained indicate that the resolution of EEG signal may not be sufficient to classify the other three hand gestures.

Despite not accounting for the variability introduced by the displacement of the electrode or the motion artifacts, our model demonstrated satisfactory performance under various measurement conditions. This suggests that stable temporal and spatial patterns in EEG signals may offer a degree of resilience to common sources of signal degradation [95].

Due to the limited dataset, balanced for each gesture with oversampling, the models used were carefully observed to determine the possibility of the occurrence of overfitting by comparing the accuracies achieved for the different training and testing sets. However, based on the large differences between the performance of individuals and gestures, it may be necessary to personalize the model.

In the study presented in this article, the detection of dependencies that may occur across all channels is not yet fully understood. Standard classifiers consistently performed worse than ANN, indicating the superiority of the latter and the strong potential for its use in BCI applications. The low F1 score observed in the results presented may be due to the limited number of training examples, algorithmic and data instability, and the high variability in EEG signals influenced by the characteristics of individual patients. It should be noted that the CNN + LSTM model worked correctly from a technical standpoint, but was unable to generalize to new cases given the limited dataset. It is also worth noting that the neural network trained on extracted features achieved better results than when using the time-windowed signal after filtering.

During the experiments, it was observed that, from a user’s perspective—particularly individuals with limited motor abilities for whom BCI could have real-world applications—the presented approaches could achieve satisfactory performance. However, its personalized mode capabilities should be considered. In other words, the system would need to be trained separately for each individual, which could prolong the calibration process and limit user convenience.

## 5. Conclusions

Applied ANNs architecture demonstrate higher effectiveness compared to standard classifiers. This may be due to the complexity of brain-derived signals. Therefore, future research should place strong emphasis on the development of neural network models capable of detecting the desired phenomena from brain-generated signals, while also offering adaptability to individual patients. It would be worth considering solutions that allow the classifier to be continuously fine-tuned during use, which would positively impact its performance by enabling adaptation to individual traits and the environment in which the patient operates.

To enhance the practical applicability of the presented methods, it would be necessary in the future to collect a significantly larger and more diverse dataset, including resting-state periods as well as pre- and post-gesture phases. This approach would not only improve the accuracy of movement initiation detection, but also leverage dynamic changes in brain activity to enhance model performance. Furthermore, techniques such as transfer learning or domain adaptation could enable initial training on a large general dataset, followed by rapid user-specific adaptation using minimal samples. Higher-order statistics derived from instantaneous frequency and amplitude may yield promising results in the case of motor execution, which should be further investigated on a larger dataset. It would also be advisable to evaluate the model’s behavior by comparing gesture execution signals with resting state (baseline) signals. In addition, performance should be assessed on a dominant hand-only dataset to isolate motor-specific neural patterns. Finally, a comprehensive comparative study is needed to validate these methods for individual applications.

Despite these limitations, the experimental results demonstrate that high classification accuracy can be achieved using EEG signals elicited by hand gestures. This represents a significant advance toward the development of simple and responsive brain–computer interfaces for patients who require assistive technologies. However, creating a universal model that performs well in diverse users remains a challenge due to the limited number of trial runs and the inherent variability of EEG signals between individuals. 

## Figures and Tables

**Table 1:** Assessment of classification.

Classifier	Variant	Accuracy [%]	F1 Score [%]
HNN	1st with feature extraction (bandpower) for all channels	74.56	22.77
LDA		50.12	22.04
SVM		67.82	25.33
HNN	1st without feature extraction for all channels	74.77	14.08
**HNN**	2nd with feature extraction (IF + IA) for all channels	**81.91**	**36.89**
LDA		73.08	18.35
SVM		75.75	17.78
HNN	2nd without feature extraction for all channels	76.36	16.80

## Data Availability

The data presented in this study are available on request from the corresponding author.
